# An Alpha-Catulin Homologue Controls Neuromuscular Function through Localization of the Dystrophin Complex and BK Channels in *Caenorhabditis elegans*

**DOI:** 10.1371/journal.pgen.1001077

**Published:** 2010-08-26

**Authors:** Linu S. Abraham, Hyun J. Oh, Feyza Sancar, Janet E. Richmond, Hongkyun Kim

**Affiliations:** 1 Department of Cell Biology and Anatomy, The Chicago Medical School, Rosalind Franklin University of Medicine and Science, North Chicago, Illinois, United States of America; 2 Department of Biological Sciences, University of Illinois at Chicago, Chicago, Illinois, United States of America; Harvard University, United States of America

## Abstract

The large conductance, voltage- and calcium-dependent potassium (BK) channel serves as a major negative feedback regulator of calcium-mediated physiological processes and has been implicated in muscle dysfunction and neurological disorders. In addition to membrane depolarization, activation of the BK channel requires a rise in cytosolic calcium. Localization of the BK channel near calcium channels is therefore critical for its function. In a genetic screen designed to isolate novel regulators of the *Caenorhabditis elegans* BK channel, SLO-1, we identified *ctn-1*, which encodes an α-catulin homologue with homology to the cytoskeletal proteins α-catenin and vinculin. *ctn-1* mutants resemble *slo-1* loss-of-function mutants, as well as mutants with a compromised dystrophin complex. We determined that CTN-1 uses two distinct mechanisms to localize SLO-1 in muscles and neurons. In muscles, CTN-1 utilizes the dystrophin complex to localize SLO-1 channels near L-type calcium channels. In neurons, CTN-1 is involved in localizing SLO-1 to a specific domain independent of the dystrophin complex. Our results demonstrate that CTN-1 ensures the localization of SLO-1 within calcium nanodomains, thereby playing a crucial role in muscles and neurons.

## Author Summary

Calcium ions are essential for many physiological processes, including neurosecretion and neuronal and muscle excitation. Paradoxically, abnormal accumulation of calcium ions is associated with cell death and has been documented as an early event in muscle and neural degenerative diseases. One mechanism to avoid detrimental calcium accumulation is to link the calcium increase with activation of calcium-dependent potassium ion channels, thereby reducing cell excitability and preventing further calcium influx. This negative feedback requires these potassium channels to be localized in close proximity to sites of calcium entry. In a *Caenorhabditis elegans* genetic screen, we identified α-catulin, known as a cytoskeletal regulatory protein in mammals, important for the localization of calcium-dependent potassium channels in both muscles and neurons. In muscle, α-catulin controls the localization of the dystrophin complex, a multimeric protein complex implicated in muscular dystrophy. The dystrophin complex in turn tethers the calcium-dependent potassium channels near calcium channels. In neurons, the α-catulin-mediated localization of the potassium channels is independent of the dystrophin complex. Lack of α-catulin results in mislocalization of the potassium channels, and in turn causes defects in neuromuscular function. Our results support the idea that cytoskeletal proteins function as anchor molecules that localize ion channels to specific cellular domains.

## Introduction

Precise control of membrane excitability, largely determined by ion channels, is of utmost importance for neuronal and muscle function. The regulation of ion channel localization, density and gating properties thus provides an effective way to control the excitability within these cells [1]. Indeed, the localization and gating properties of ion channels are often regulated or modified by cytoskeletal and signaling proteins, or auxiliary ion channel subunits expressed in a cell-type specific manner [2]. Potassium channels are critical in determining the excitability of cells, because potassium ions are dominant charge carriers at the cell resting potential. Among potassium channels, the large conductance, voltage- and calcium-dependent potassium BK channels (also called SLO-1 or Maxi-K) are uniquely gated by coincident calcium signaling and membrane depolarization [3], [4]. This feature of BK channels provides a crucial negative feedback mechanism for calcium-induced functions, and plays an important role in determining the duration of action potentials [3]. BK channels are widely expressed in a variety of cell types and are implicated in many physiological processes, including the regulation of blood pressure [5], neuroendocrine signaling [6], smooth muscle tone [7], and neural network excitability [8], [9].

Mounting evidence indicates that BK channels can interact with a variety of proteins that modulate channel function, or control membrane trafficking. For example, the *Drosophila* BK channel, dSLO, interacts with SLO binding protein (slob), which in turn modulates the channel gating properties [10]. Similarly, mammalian BK channels associate with auxiliary beta subunits that influence channel activation time course and voltage-dependence [11]. In yeast two hybrid screens, the cytoplasmic C-terminal tail of mammalian BK channels has been shown to interact with several proteins, including cytoskeletal elements, such as actin-binding proteins [12], [13] and a microtubule-associated protein [14]. These cytoskeletal proteins are partially co-localized with BK channels, and appear to increase cell surface expression of BK channels in cultured cells [12], [13]. However, it remains to be determined whether these proteins have any role in controlling the localization of BK channels to specific areas of the plasma membrane *in vivo*. Robust activation of BK channels requires higher intracellular calcium concentrations (>10 µM), which only occur in the immediate vicinity of calcium-permeable channels [4]. Hence, the localization of BK channels to specific areas (i.e. calcium nanodomains) where calcium-permeable ion channels are located is physiologically important for BK channel activation.

In *C. elegans*, loss-of-function mutations in *slo-1* partially compensate for the synaptic release defects of *C. elegans* syntaxin (*unc-64*) mutants [15] and lead to altered alcohol sensitivity [16]. Recent studies in *C. elegans* have also implicated SLO-1 in muscle function [17]. *slo-1* mutants display an exaggerated anterior body angle, referred to as the head-bending phenotype that is shared by mutants that are defective in the *C. elegans* dystrophin complex [18]–[20]. Recent evidence that the *C. elegans* dystrophin complex interacts with SLO-1 channels via SLO-1 interacting protein, ISLO-1, explains this phenotypic overlap [21]. However, *C. elegans* dystrophin complex mutants do not appear to alter the biophysical properties of BK channels *per se* [17]. Similarly, ISLO-1 does not modify SLO-1 channel properties [21]. Rather, ISLO-1 tethers SLO-1 near the dense bodies of muscle membranes, where L-type calcium channels (EGL-19) are localized [21]. Consequently, defects in the dystrophin complex or ISLO-1 cause a large reduction in SLO-1 protein levels in muscle membrane, which in turn causes muscle hyper-excitability leading to enhanced intracellular calcium levels. This perturbation of calcium homeostasis has been postulated to be one of the first steps in the degenerative muscle pathogenesis associated with disruption of the dystrophin complex [22].

In this study, we performed a forward genetic screen to identify additional genes responsible for SLO-1 localization and function in *C. elegans*. We identified *ctn-1*, an orthologue of α-catulin, as a novel gene that controls SLO-1 localization and function in muscles and neurons. Our analysis showed that *ctn-1* uses different strategies to localize SLO-1 in these two cell types. In muscles, CTN-1 utilizes the dystrophin complex to localize SLO-1 near L-type calcium channels via ISLO-1. In neurons, CTN-1 localizes SLO-1 independent of the dystrophin complex.

## Results

### A genetic screen for suppressor mutants of gain-of-function *slo-1* identifies genes that interact with the dystrophin gene

**Figure 1:**
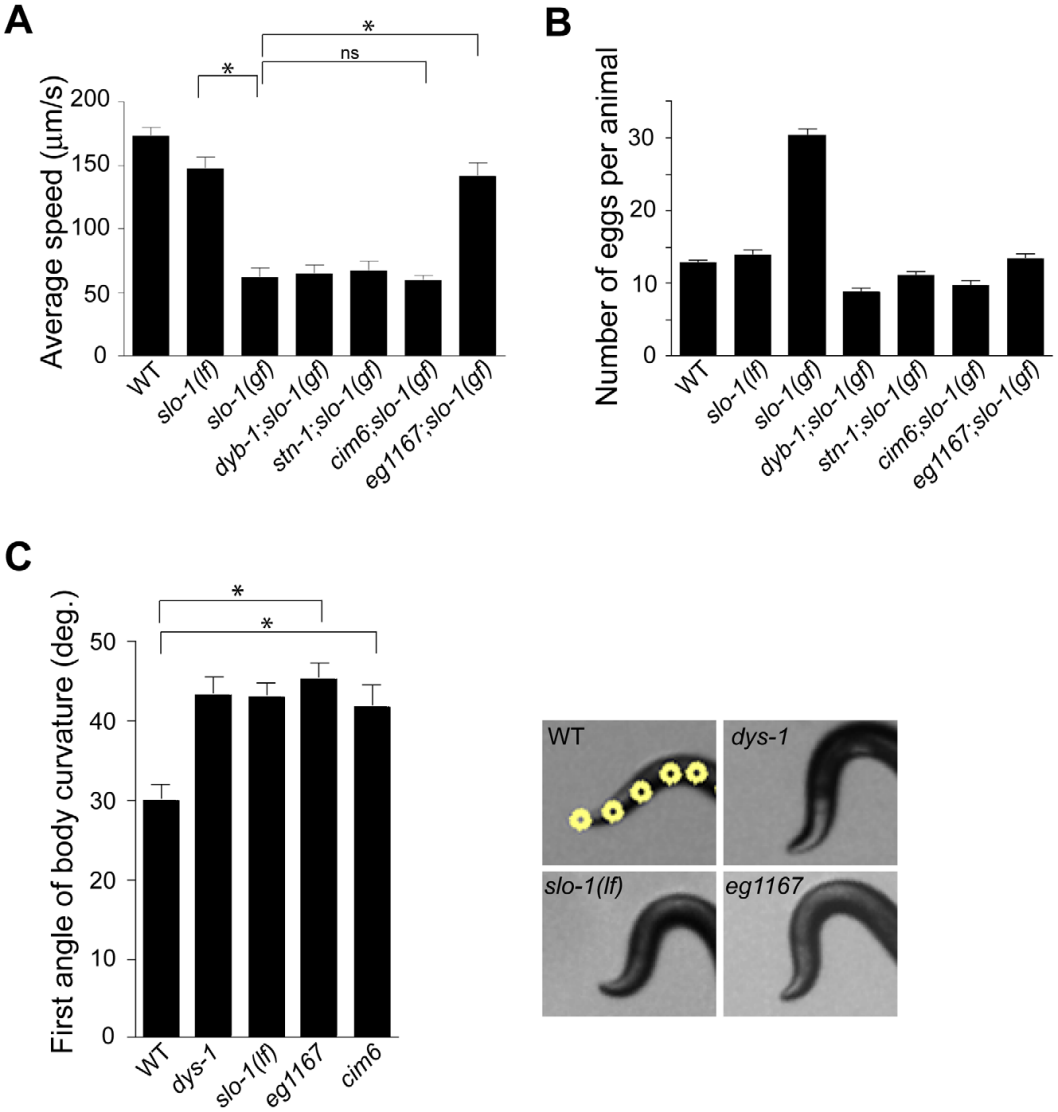
A genetic screen for *slo-1(gf)* suppressor mutants yields genes encoding components of the dystrophin complex and a novel gene. (A) The average speed of mutants identified in a genetic screen for suppressors of *slo-1(gf)*. *dyb-1*, a dystrobrevin homolog; *dys-1*, a dystrophin homolog; *stn-1*, a syntrophin homolog. Error bars represent s. e. m. (*n*>10). Asterisks represent significant difference (*P*<0.05). (B) The number of eggs retained in uteri of *slo-1(gf)* suppressor mutants. Error bars represent s. e. m. Data points between *slo-1(gf)* and all of other strains are significantly different (*P*<0.001). (C) Quantitative analysis for the first angle of head bending. Each data set for the first angle is significantly different from that of wild-type animals (*P*<0.01). Yellow dots indicate the five most anterior of the 13 midline points for a wild-type animal (See also Materials and Methods). Error bars represent s. e. m. (*n* = 10).

Loss-of-function *slo-1* mutants exhibit a jerky locomotion and head bending phenotype [15]. By contrast, gain-of-function *slo-1* mutants exhibit sluggish movement combined with low muscle tone [16]. When *slo-1(gf)* mutant animals are mechanically stimulated, they fail to make a normal forward movement, and tend to curl ventrally (Video S1). To identify genes that regulate *slo-1* function, we performed a forward genetic screen to isolate mutants that suppress the phenotypes of the *slo-1*(*ky399*) gain-of-function mutant. Based on a previous genetic study [21], suppressor genes were expected to encode *slo-1*, components of the dystrophin complex, as well as novel proteins that control neuronal or muscular function of SLO-1. As expected, several loss-of-function alleles of *slo-1* were isolated. In addition to these intragenic suppressors, several mutants could be segregated away from *slo-1(gf)* (Figure 1A and Video S1) and exhibited the head bending phenotype. Genetic mapping and complementation testing determined that these extragenic suppressors include *dyb-1* and *stn-1* which encode two homologous components of the dystrophin complex, dystrobrevin and syntrophin respectively. Additionally we isolated *cim6* and *eg1167* suppressors that represent novel genes. Compared to *slo-1*(*ky399*) and *cim6;slo-1*(*ky399*) mutants, *eg1167*;*slo-1*(*ky399*) mutants exhibited a profound improvement in the locomotion speed (Figure 1A).

It was previously observed that *slo-1(gf)* mutants retain significantly more eggs than wild-type animals due to low activity of the egg-laying muscles [16]. We found that suppressor mutants abolish an egg laying defect of *slo-1(gf)* mutants and retain eggs in uteri at levels similar to wild-type animals (Figure 1B).

### *ctn-1* encodes an α-catulin orthologue that has homology to α-catenin and vinculin

**Figure 2:**
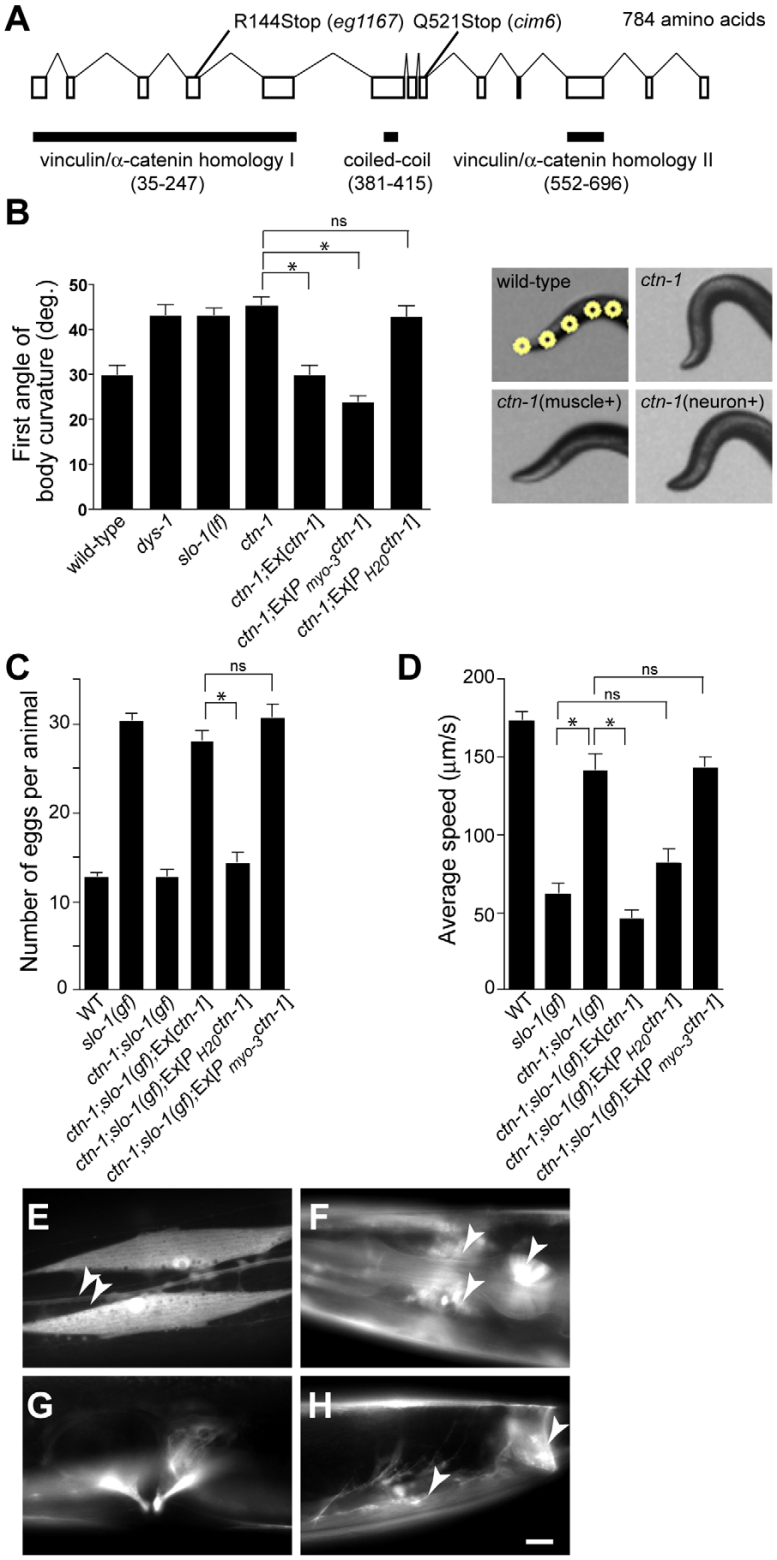
*ctn-1*, an α-catulin homologue, has two distinct roles in mediating SLO-1 function. (A) The gene structure of *ctn-1*. Our genome analysis indicates that unlike WormBase annotation (WS210) *ctn-1* consists of 13 exons and is predicted to encode a 784 amino-acid protein (Figure S1). The homology regions with α-catenin/vinculin I (homologous to the N-terminal talin/α-actinin-binding region of vinculin, 213 amino acids), α-catenin/vinculin II (homologous to the C-terminal F-actin/inositol phospholipids-binding region of vinculin, 145 amino acids) and the coiled-coil domain (35 amino acids) are depicted at the bottom of the gene structure. The mutation sites for two different alleles (*eg1167* and *cim6*) are shown on the top of the gene structure. The predicted amino acid sequence is available in Figure S1. (B) Rescue of the head bending phenotype with a variety of *ctn-1* constructs. Ex[*ctn-1*] represents the transgene carrying the genomic *ctn-1* DNA extrachromosomal array. Ex[P*_myo_*_-*3*_ *ctn-1*] represents the muscle-specific *myo-3* promoter-driven *ctn-1* transgene, whereas Ex[P*_H20_ctn-1*] represents the neuron-specific *H20* promoter-driven *ctn-1* transgene. Error bars represent s. e. m. (*n* = 10). Single asterisks indicate significant difference between two groups (*P*<0.001, unpaired two-tailed t-test) whereas ns indicates no significant difference. (C) Rescue of the defect in egg-laying muscle with a variety of *ctn-1* constructs. Error bars represent s. e. m. (*n* = 15). Single asterisks indicate significant difference between two groups (*P*<0.001, unpaired two-tailed t-test) whereas ns indicates no significant difference. (D) Rescue of the average locomotory speed with a variety of *ctn-1* constructs. Error bars represent s. e. m. (*n*>10). Single asterisks indicate significant difference between two groups (*P*<0.001, unpaired two-tailed t-test) whereas ns indicates no significant difference. (E–H) The expression pattern of a *ctn-1* promoter-tagged GFP reporter. Expression in (E) body wall muscles and the ventral cord neurons (arrowheads), (F) nerve ring (arrowheads) and pharyngeal muscle (arrow), (G) egg-laying muscle, and (H) enteric (arrow) and sphincter (arrowhead) muscle. Scale bar, 10 µm.

To understand the role of novel genes in *slo-1* function, we pursued the identification of genes that mapped to chromosomal locations neither previously implicated in BK channel function, nor encoding known components of the dystrophin complex. Two mutations, *cim6* and *eg1167*, both mapped to the left side of chromosome I and failed to complement each other for head bending, suggesting that these two mutations represent alleles of the same gene. Our quantitative analysis for locomotion and egg laying phenotypes showed that the locomotion speed of *eg1167;slo-1(gf)* was higher than that of *cim6;slo-1(gf)* whereas egg laying was comparable in both strains (Figure 1A and 1B). We further mapped *eg1167* to a 250 kb interval and rescued the phenotype of *eg1167* by generating transgenic animals with the fosmid WRM0621cC01 (Figure S1). Next, we rescued the head bending phenotype of *eg1167* with a transgene consisting of the *ctn-1* gene (Y23H5A.5) and approximately 4 kb upstream of the translation initiation codon (Figure 2A and 2B). The same transgene caused *eg1167;slo-1(gf)* double mutants to revert to the *slo-1(gf)* phenotype, displaying sluggish movement and retention of late-staged eggs in uteri (Figure 2C and 2D). These results indicate that a genetic defect in *ctn-1* is responsible for suppression of the *slo-1(gf)* phenotypes.

The *ctn-1* gene is orthologous to mammalian α-catulin (39.4% identity to human α-catulin), and is named on the basis of sequence similarity to both α-catenin and vinculin (Figure 2A) [23]. Vinculin and α-catenin are membrane-associated cytoskeletal proteins found in focal adhesion plaques and cadherens junctions. In *C. elegans*, vinculin (DEB-1) is localized to the dense bodies of body wall muscle and is essential for attachment of actin thin filaments to the sarcolemma [24], whereas α-catenin (HMP-1) is localized to hypodermal adherens junctions and is essential for proper enclosure and elongation of the embryo [25]. Based on its homology to vinculin/α-catenin and the localization of mammalian α-catulin [26], CTN-1 is likely to interact with other cytoskeletal proteins, which may in turn affect SLO-1 function. Additionally, the *ctn-1* gene encodes a predicted coiled-coil domain. Such a coiled-coil domain mediates the interaction between dystrophin and dystrobrevin [27], two components of the dystrophin complex, although we do not know if the coiled-coil domain of CTN-1 is important for the interaction with these proteins (Figure 2A).

We determined nucleotide sequence of the predicted exons and exon-intron boundaries of the *ctn-1* gene in *eg1167* and *cim6*. The mutation sites found in both alleles create translation-termination codons (R144>STOP in *eg1167*, Q521>STOP in *cim6*) (Figure 2A). *eg1167* exhibits complete suppression of *slo-1*(*gf*) phenotypes (see below) and is hence considered as a severe loss-of-function or null allele. All subsequent experiments were carried out with *eg1167*, unless mentioned otherwise. Although both *eg1167* and *cim6* mutants alone exhibit the head-bending phenotype, they differ with respect to suppression of *slo-1(gf)* phenotypes. Whereas *ctn-1(eg1167)* suppresses all aspects of the *slo-1(gf)* phenotype, *ctn-1*(*cim6*) completely suppresses the egg-laying defect of *slo-1(gf)* (Figure 1B), but not the locomotory defect (Figure 1A). These results suggest that the C-terminal third of CTN-1 is required for normal egg laying and head bending, but is not necessary to mediate the locomotion speed defect of *slo-1(gf)* mutants.

To elucidate the function of CTN-1, we examined the expression pattern of the *ctn-1* gene using a *ctn-1* promoter-tagged GFP reporter (Figure 2E–2H). We observed GFP fluorescence in body wall muscles, pharyngeal muscle, egg-laying muscle and enteric muscle of transgenic animals as well as in most, if not all, neurons of the nerve ring and ventral nerve cord.

### CTN-1 has two distinct functions in neurons and muscles

Based on the *ctn-1* expression pattern and the phenotypic differences between *eg1167* and *cim6*, we investigated whether the head-bending phenotype and the suppression of sluggish movement of *slo-1(gf)* mutants are separable by expressing *ctn-1* minigenes under the control of either muscle- or neuron-specific promoters in *ctn-1* and *ctn-1;slo-1(gf)* mutant animals. Muscle, but not neuronal, expression of *ctn-1* rescued the head-bending phenotype of the *ctn-1* mutant (Figure 2B and Figure S1C). These results are consistent with previous reports that the head-bending phenotype is due to perturbations in muscle function [17]–[19]. Furthermore, muscle expression of *ctn-1* in *ctn-1*;*slo-1(gf)* mutants resulted in egg retention to the level observed in *slo-1(gf)* mutants, whereas neuronal expression of *ctn-1* did not alter the number of eggs retained in the uteri of *ctn-1*;*slo-(gf)* mutants (Figure 2C). Conversely, neuronal expression of *ctn-1* in *ctn-1;slo-1(gf)* mutants reverted the seemingly normal locomotion of *ctn-1;slo-1(gf)* to the sluggish, uncoordinated locomotion of the *slo-1(gf)* mutant, whereas muscle expression of *ctn-1* did not (Figure 2D). These results indicate that the sluggish, uncoordinated locomotory phenotype of *slo-1(gf)* mutants comes from presynaptic depression, but not from direct suppression of muscle excitability. Together with the allele specific phenotypic differences indicating different regions of CTN-1 are required for normal locomotory speed and head bending, these results suggest that CTN-1 uses two distinct mechanisms for mediating SLO-1 function in muscle and neurons by interacting with different sets of genes.

### CTN-1 controls the integrity of the dystrophin complex and the localization of SLO-1 in muscle

**Figure 3:**
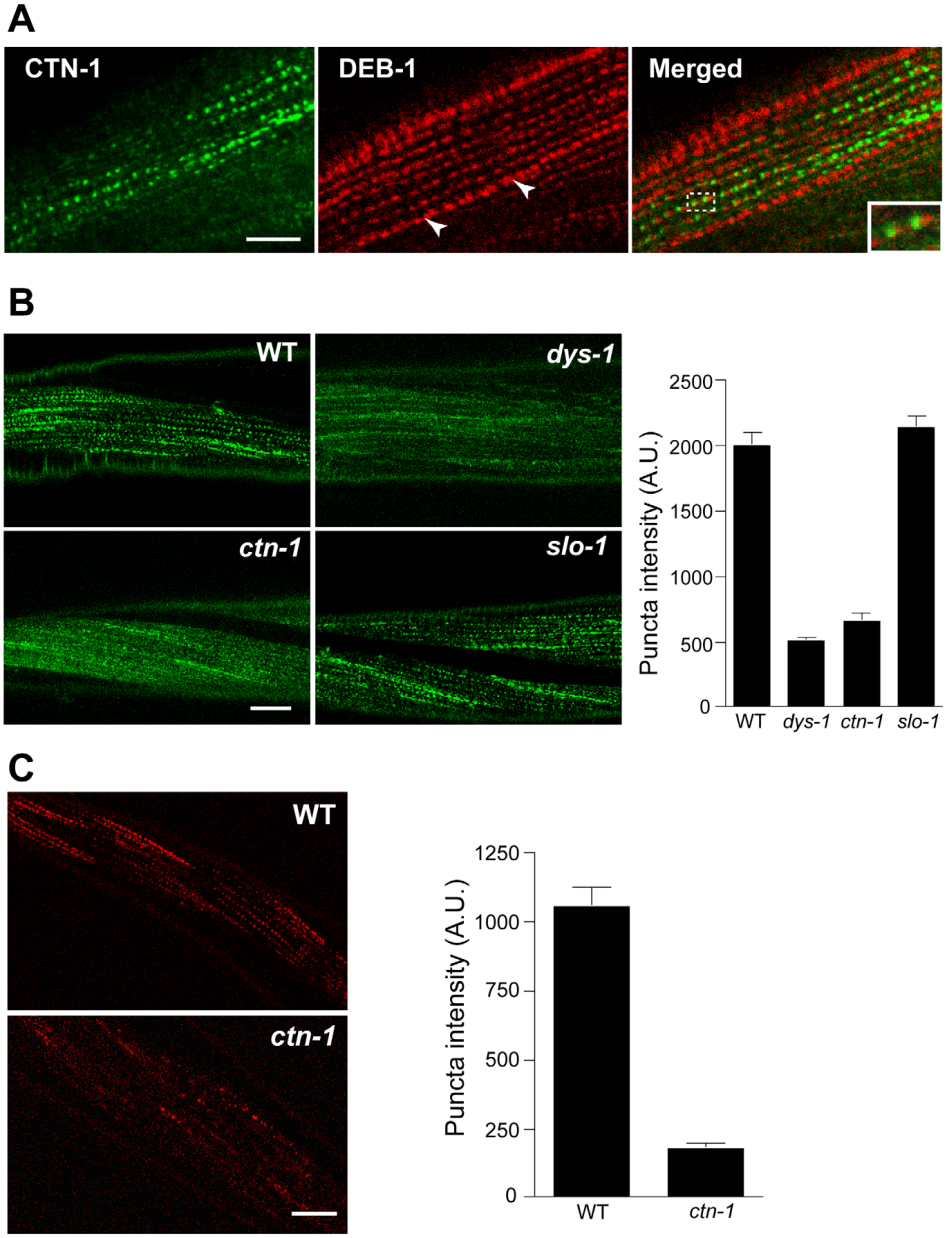
*ctn-1* mutation disrupts normal localization of the dystrophin complex and ISLO-1. (A) An integrated transgenic line expressing the lowest level of GFP-tagged CTN-1 was used for staining with anti-GFP (CTN-1, *green*) and anti-vinculin/DEB-1 (DEB-1, *red*) antibodies. Dashed box area is enlarged in the bottom left of the panel (*Merged*) to show detail. Arrowheads indicate the attachment plaques that adhere tightly adjacent muscle cells. Scale bar, 10 µm. (B) Transgenic animals expressing integrated GFP-tagged SGCA-1 were used to analyze the localization of SGCA-1 in wild-type (WT), *dys-1 ctn-1* and *slo-1* animals. Scale bar, 10 µm. The graph shows quantified puncta intensities. Error bars, s.e.m. Wild-type vs. *dys-1* (*P*<0.01), Wild-type vs. *ctn-1* (*P*<0.01), Wild-type vs. *slo-1* (P>0.05). (C) Transgenic animals expressing integrated mCherry-tagged ISLO-1 were used for analyzing the localization of ISLO-1 in wild-type (WT) and *ctn-1* animals. Scale bar, 10 µm. The graph shows quantified puncta intensities. Error bars, s.e.m. Wild-type vs. *ctn-1* (*P*<0.0001).

Most, if not all, of the mutants that exhibit the head bending phenotype have a defect in either a component of the dystrophin complex or proteins that interact with the dystrophin complex [17]–[19]. The dystrophin complex is localized near muscle dense bodies [21]. Because *ctn-1* mutants exhibit the head bending phenotype, we determined the subcellular localization of CTN-1 using a GFP-tagged CTN-1 transgene, which rescues the head bending phenotype (data not shown). GFP::CTN-1 exhibited a punctate expression pattern that resembled that of the dense bodies (Figure 3A). To further define the localization of CTN-1, we stained GFP-tagged CTN-1 transgenic animals with GFP antibodies and vinculin/DEB-1 antibodies that recognize the attachment plaque and dense bodies. CTN-1::GFP is localized in close proximity to, or partially colocalized with, vinculin/DEB-1 in dense bodies, but not in the attachment plaques, indicating that CTN-1 is localized near dense bodies (Figure 3A). This expression pattern of CTN-1, along with the head bending phenotype of *ctn-1* mutants, prompted us to examine whether the *ctn-1* mutation disrupts the integrity of the dystrophin complex. We compared the expression pattern of a component of the dystrophin complex, SGCA-1 (an α-sarcoglycan homolog) in wild-type, *dys-1*, *slo-1* and *ctn-1* animals using a GFP-tagged SGCA-1 that rescues the head bending phenotype of *sgca-1* mutants [21] (Figure 3B). GFP::SGCA-1 exhibited a punctate expression pattern in the muscle membrane of wild-type and *slo-1* mutant animals. By contrast, GFP puncta were greatly diminished in *dys-1* and *ctn-1* mutants. These results indicate that *ctn-1* is critical for maintaining the dystrophin complex near the dense bodies.

We previously demonstrated that ISLO-1 interacts with STN-1 through a PDZ domain-mediated interaction, thereby linking SLO-1 to the dystrophin complex [21]. Because we failed to observe a component of the dystrophin complex in the muscle membrane of *ctn-1* mutants, we examined mCherry-tagged ISLO-1 in the muscle membrane of wild-type and *ctn-1* mutant animals. The punctate mCherry::ISLO-1 fluorescence was observed in wild-type muscle membranes, but was greatly reduced in *ctn-1* mutant (Figure 3C). These results further strengthen the notion that CTN-1 is required for maintaining the integrity of the dystrophin complex.

**Figure 4:**
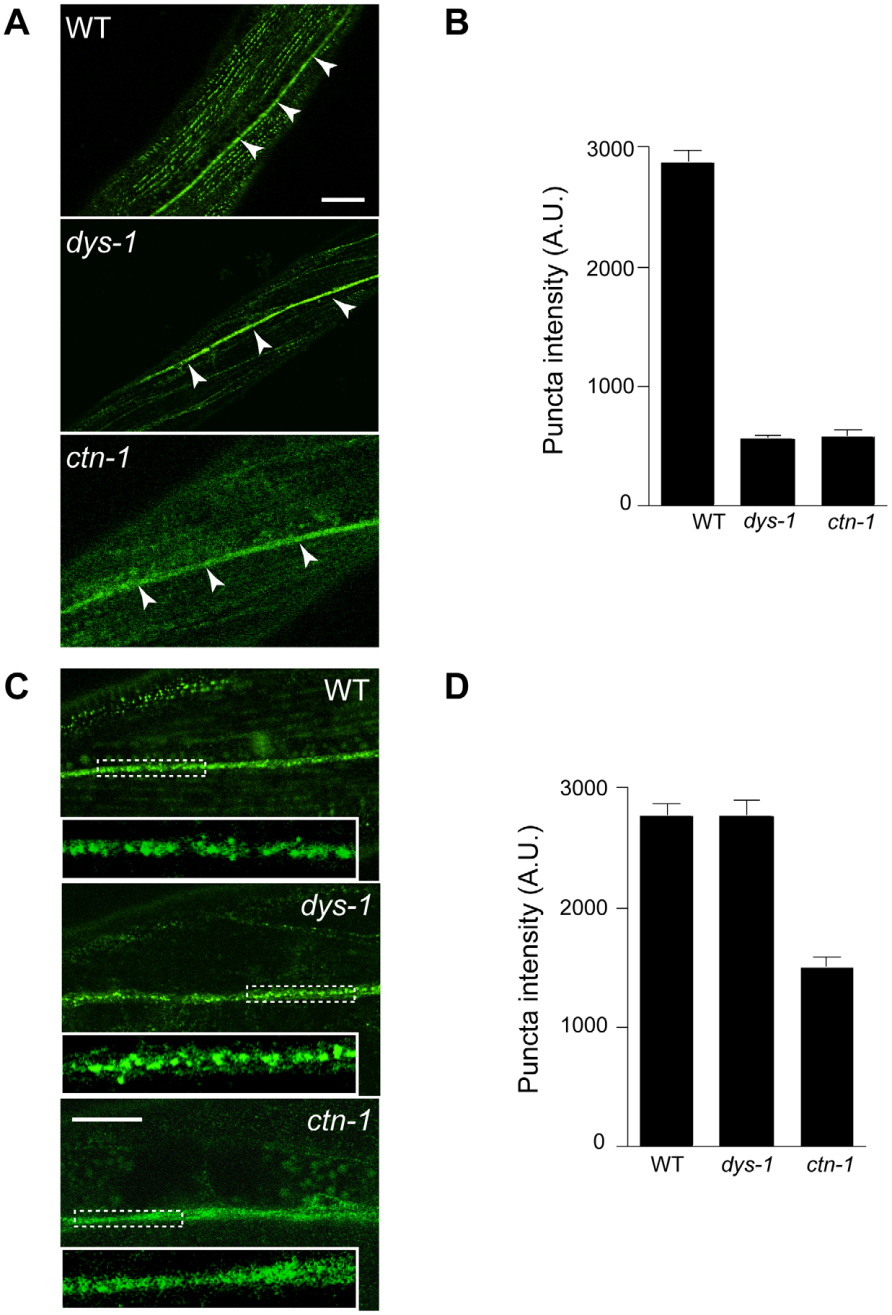
*ctn-1* mutation impairs normal localization of SLO-1 in muscles and neurons. The same integrated array, SLO-1::GFP, was used for this analysis in different genetic backgrounds. (A–B) Muscular localization of SLO-1::GFP in wild-type, *dys-1* and *ctn-1* animals. Arrowheads represent the ventral (or dorsal) nerve cords. (B) The graph showing quantification of puncta intensities. Error bars, s.e.m. Wild-type vs. *dys-1* or *ctn-1* (*P*<0.0001). Scale bar, 10 µm. (C–D) Neuronal localization of SLO-1::GFP in wild-type, *dys-1* and *ctn-1* animals (See also Figure S2). Regions of the ventral nerve cord (*dashed boxes*) are enlarged in the bottom left of each panel to show detail. (D) The graph showing quantification of puncta intensities. Error bars, s.e.m. Wild-type vs. *dys-1* (*P*>0.05), Wild-type vs. *ctn-1* (*P*<0.0001). Scale bar, 10 µm.

Based on the genetic interaction between *ctn-1* and *slo-1*, and the observation that the integrity of the dystrophin complex and ISLO-1 localization are disrupted in *ctn-1* mutants, we hypothesized that CTN-1 regulates the localization of SLO-1 in muscle. To test this hypothesis, we examined the localization of GFP-tagged SLO-1 in muscles of wild-type, *dys-1*, and *ctn-1* animals (Figure 4A and 4B). The punctate SLO-1::GFP expression pattern in the muscle membrane of wild-type animals was greatly diminished in the muscles of either *dys-1* or *ctn-1* mutant. Interestingly, the protein levels of SLO-1::GFP were not significantly different in wild-type, *dys-1* and *ctn-1* animals (Figure S2B), indicating that mislocalized SLO-1 does not necessarily undergo degradation. The mislocalization of SLO-1 in *dys-1* mutants is consistent with the requirement of the dystrophin complex for ISLO-1 localization [21]. These results further indicate that CTN-1 stabilizes or maintains the punctate muscle expression of SLO-1::GFP in a dystrophin complex-dependent manner.

### CTN-1 regulates presynaptic release by controlling the localization of SLO-1

In mammals, BK channels are found in neuronal somata, dendrites and presynaptic terminals [28], [29]. An immunoelectron microscopy study indicates that BK channels are not homogeneously distributed in neurons, but are clustered, presumably near calcium channels [30]. We addressed whether SLO-1 is evenly distributed or clustered in *C. elegans* neurons by examining SLO-1::GFP. Wild-type animals displayed patches of fluorescence along the ventral nerve cord or near cell bodies under high magnification (Figure 4C and 4D, Figure S2). Tissue-specific rescue experiments demonstrated that *ctn-1* mediates SLO-1 function in neurons independent of the dystrophin complex (Figure 2D). Therefore, we compared neuronal SLO-1::GFP expression in *dys-1* and *ctn-1* mutant animals. The clustered GFP expression observed along the ventral cord of both wild-type and *dys-1* mutant animals contrasted with the uniform GFP localization in *ctn-1* mutants (Figure 4C and 4D). These results indicate that *ctn-1* mutation disrupts the neuron-specific clustering of SLO-1::GFP independent of the dystrophin complex.

**Figure 5:**
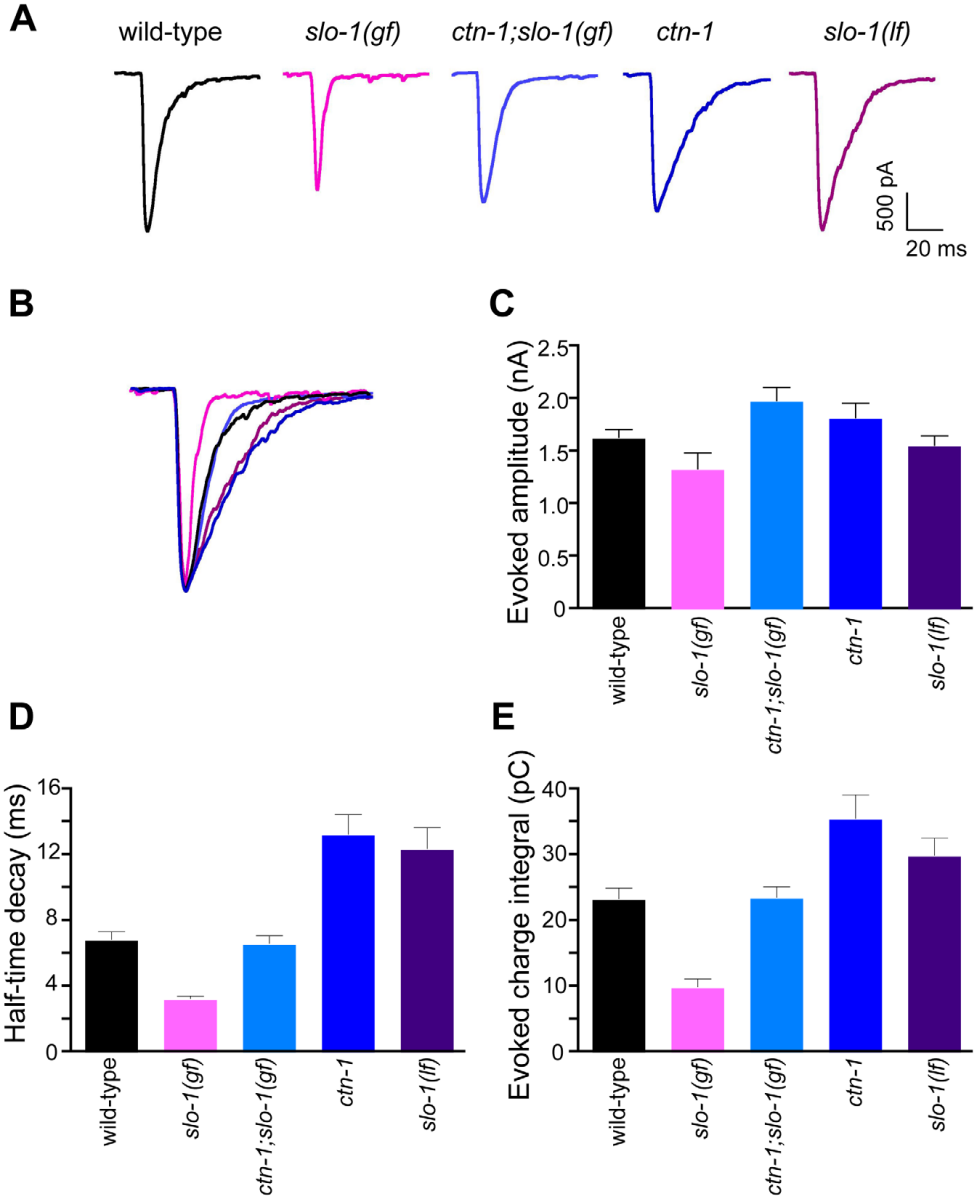
*ctn-1* mutation suppresses defects of *slo-1(gf)* evoked synaptic responses at the neuromuscular junctions. (A) Representative evoked current responses from wild-type, *slo-1(gf)*, *ctn-1;slo-1(gf)*, *ctn-1* and *slo-1(lf)* animals. (B) Normalized evoked current responses from (A). (C) Evoked amplitude response. Wild-type (n = 31), *slo-1(gf)* (n = 15), *ctn-1;slo-1(gf)* (n = 12), *ctn-1* (n = 16), *slo-1(lf)* (n = 21). There is no significant difference between wild-type and each genotype used (*P*>0.05). (D) Half-time decay. Wild-type *vs. slo-1(gf)*, *P*<0.05; wild-type *vs. ctn-1;slo-1(gf)*, *P*>0.05; wild-type *vs. ctn-1*, *P*<0.05; wild-type *vs. slo-1*, *P*<0.01. (E) Evoked charge integral. Wild-type *vs. slo-1(gf)*, *P*<0.01.

SLO-1 contributes to the repolarization of the synaptic terminal following neuronal stimulation, thereby terminating neurotransmitter release. Consequently loss-of-function *slo-1* mutants are hypersensitive to the paralyzing effects of aldicarb, an acetylcholinesterase inhibitor, a phenotype indicative of enhanced acetylcholine release. Consistent with this interpretation, electrophysiological recordings from neuromuscular junctions of *slo-1* loss-of-function mutants exhibit prolonged evoked synaptic responses [15], [16]. If CTN-1 regulates SLO-1 localization in motor neurons and thus *slo-1* function, we would expect *ctn-1* mutants to exhibit similar pharmacological and synaptic changes. Indeed, we found that *ctn-1* mutants were hypersensitive to aldicarb compared to wild-type animals (Figure S3). To confirm this observation directly, we measured synaptic responses from the neuromuscular junctions of dissected wild-type and *ctn-1* mutant animals engineered to express channelrhodopsin-2 in motor neurons [31] (Figure 5). Evoked synaptic responses were elicited by blue light activation of channelrhodopsin-2 and recorded from voltage-clamped post-synaptic body wall muscle cells. Consistent with our pharmacological data and localization results, recordings from *ctn-1* showed prolonged evoked synaptic responses similar to those of *slo-1(lf)* mutants (Figure 5B and 5C). Furthermore, muscular expression of *ctn-1* in *ctn-1* mutant animals rescued the head-bending phenotype (Figure 2B), but did not rescue prolonged evoked synaptic responses (Figure S3B). These data strongly suggest that altered synaptic responses of *ctn-1* mutants result from a neuronal defect.

In contrast to the *slo-1(lf*) mutants, evoked responses of *slo-1(gf)* mutants were short-lived (Figure 5C), and the charge integral, a measure of total ion flux during the evoked response, was significantly reduced (Figure 5E). Our genetic analyses demonstrated that the *ctn-1* mutation suppresses the sluggish locomotory phenotype of *slo-1(gf)* mutants and disrupts SLO-1 localization (Figure 1A, Figure 4C and 4D). If this is due to loss of neuronal SLO-1(*gf*) channels, the *ctn-1* mutation should suppress the evoked response defects of *slo-1(gf)*. Consistent with this prediction, the decay time of the *ctn-1*;*slo-1(gf)* double mutants (t_1/2_ = 6.61±0.53 ms) was significantly longer than *slo-1(gf)* (t_1/2_ = 3.23±0.21 ms) (Figure 5D), and the charge integral was restored to wild-type levels (Figure 5E). Interestingly, *ctn-1* mutants did not convert the decay time of *slo-1(gf)* evoke responses to that of *slo-1(lf)*, indicating that residual SLO-1 function may be mediated by dispersed SLO-1 channels.

## Discussion

**Figure 6:**
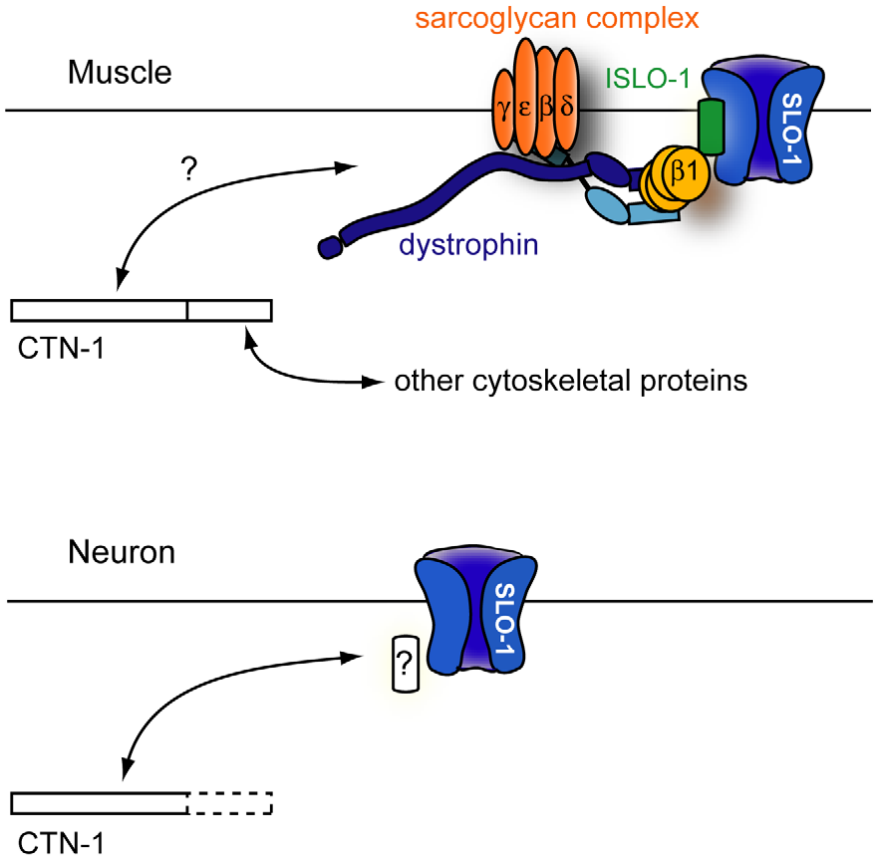
A model for CTN-1 function. In muscles, CTN-1 interacts with the dystrophin complex. ISLO-1 links the dystrophin complex to SLO-1. The N-terminal region or the coiled-coiled domain of CTN-1 is likely to interact with the dystrophin complex. The C-terminal region may interact with other cytoskeletal proteins. In neurons, CTN-1 interacts with SLO-1 through possible unknown intermediates other than the dystrophin complex.

In a genetic screen to identify novel regulators of SLO-1, we found two alleles of *ctn-1*, a gene which encodes an α-catulin orthologue. CTN-1 mediates normal bending of the anterior body through SLO-1 localization near the dense bodies of body wall muscles. CTN-1 also maintains normal locomotory speed through SLO-1 localization within neurons. Based on our data, we propose a model for *ctn-1* function in localizing SLO-1 (Figure 6). In muscles, CTN-1 interacts with the dystrophin complex. It is also possible that CTN-1 may influence the stability of another protein that directly interacts with the dystrophin complex. Loss of CTN-1 function disrupts the integrity of the dystrophin complex, thus compromising ISLO-1 and SLO-1 localization near muscle dense bodies, where L-type calcium channels are present. Disruption of SLO-1 localization is expected to uncouple local calcium increases from SLO-1-dependent outward-rectifying currents, resulting in muscle hyper-excitation. Previous studies have shown that the head bending phenotype, shared among mutants that have a defect in the dystrophin complex or its associated proteins, results from muscle hyperexcitability [17]–[19], [32]. Our data further show that this head-bending phenotype does not result from a synaptic transmission defect, but from a muscle excitation and contraction defect. In neurons, SLO-1 localization is not mediated through the dystrophin complex, suggesting that CTN-1 interacts with other proteins to localize SLO-1 to specific neuronal domains.

Why does CTN-1 use two distinct mechanisms to localize SLO-1 to subcellular regions of muscles and neurons? BK channels are functionally coupled with several different calcium channels (including voltage-gated L-type and P/Q-type calcium channels and IP3 receptors) that are localized in different subcellular regions [30], [33], [34]. Although it has not been determined whether all of these calcium channels are functionally coupled with SLO-1 in *C. elegans*, these calcium channels are distributed in different regions of neurons. For example, the L-type calcium channel (EGL-19) is mainly expressed in the cell body and the P/Q type calcium channel (UNC-2) is concentrated at the presynaptic terminals [35], [36]. A distinct set of proteins is perhaps required for SLO-1 channel localization near different calcium channels.

How CTN-1 interacts with the dystrophin complex in muscle remains to be determined. It has been suggested that mammalian α-catulin interacts with the hydrophobic C-terminus of dystrophin resulting from alternative splicing [37]. However, the *C. elegans dys-1* gene does not encode a hydrophobic C-terminus. Thus, CTN-1 may interact with a different domain of dystrophin, or with another component of the dystrophin complex. In this regard, it is noteworthy that both mammalian dystrophin and *C. elegans* DYS-1 have multiple spectrin repeat domains, and that the N-terminal region of vinculin which exhibits homology to that of α-catulin (Figure S1) is known to bind the spectrin repeat domain of α-actinin [38]. By extension, we speculate that the N-terminal region of CTN-1 may bind the spectrin repeat domain of DYS-1 directly. Alternatively, the coiled-coil domain of dystrophin, which is known to interact with the coiled-coil domain of dystrobrevin [27], may potentially bind the coiled-coil domain of CTN-1. Interestingly, CTN-1 exhibits high homology to vinculin in both the N-terminal and C-terminal regions (Figure S1B). The C-terminal region of vinculin interacts with cytoskeletal molecules or regulators (F-actin, inositol phospholipids and paxillin) in focal adhesion and adherens junctions [39]. Because the C-terminal region of CTN-1 is also necessary for normal head bending, we speculate that this C-terminal region may be important for tethering the dystrophin complex to other cytoskeletal proteins.

In mammalian striated muscle, dystrophin is enriched in costameres [40] which are analogous to *C. elegans* dense bodies. A costamere is a subsarcolemmal protein assembly that connects Z-disks to the sarcolemma, and is considered to be a muscle-specific elaboration of the focal adhesion in which integrin and vinculin are abundant. Compromised costameres have been postulated to be an underlying cause of several different myopathies [40]. It was recently shown that ankyrin-B and -G recruit the dystrophin complex to costameres [41]. Based on overall high homology of *ctn-1* to vinculin and α-catenin, we speculate that CTN-1 similarly interacts with cytoskeletal proteins in the dense bodies, and links the dystrophin complex to the dense bodies.

Another intriguing conclusion from our data is that loss of CTN-1 does not completely abolish SLO-1 function. Complete abolishment of SLO-1 function in *ctn-1* mutant should alter the decay time for evoked synaptic responses of *ctn-1*;*slo-1(gf)* to the same degree as *slo-1(lf)* mutants, rather than to that of wild-type animals (Figure 5D). Mutants including *slo-1(gf)*, that have defects in neural activation or membrane depolarization, are reported to cause *str-2*, a candidate odorant receptor gene, to be expressed in both AWC olfactory neurons whereas wild-type animals express *str-2* in only one of the AWC pair [42]. We find that *ctn-1* mutation does not suppress the misexpression of *str-2* in both AWC neurons in *slo-1(gf)* mutants, suggesting that *ctn-1* mutations do not completely abolish SLO-1 function (unpublished observations, HK). It is thus likely that the defect in SLO-1 localization in *ctn-1* mutants makes it less responsive to local calcium nanodomains found at presynaptic terminals and dense bodies, but still able to respond to depolarization-induced global calcium increases, albeit at a lower level.

In conclusion, we have identified *ctn-1*, a gene encoding the *C. elegans* homolog of α-catulin, and demonstrated that CTN-1 mediates SLO-1 localization in muscles and neurons by dystrophin complex-dependent and -independent mechanisms, respectively. How SLO-1 is localized to certain neuronal domains will require further screening of *slo-1(gf)* suppressor mutants. Given that proteins affecting components of the dystrophin complex are likely to contribute to the pathogenesis of muscular dystrophy, α-catulin is a candidate causal gene for a form of muscular dystrophy in humans.

## Materials and Methods

### Strains and genetics

The genotypes of animals used in this study are: N2 (wild-type), CB4856, *dys-1(eg33) I*, *stn-1(tm795) I*, *ctn-1(eg1167) I*, *ctn-1(cim6) I*, *slo-1(eg142) V*, *slo-1(ky399gf) V* and *sgca-1(tm1232) X*. The following transgenes were used in this study: *cimIs1*[*slo-1a::GFP*, *rol-6(d)*] [21], *cimIs5*[*mCherry::islo-1*, *ofm-1::GFP*] [21], *zxIs6*[*unc-17::chop-2(H134R)-yfp*; *lin-15(+)*] [31], *cimIs6*[*GFP::sgca-1*, *rol-6(d)*], *cimEx5*[*ctn-1*, *ofm-1::*GFP], *cimIs7*[*GFP::ctn-1*, *rol-6(d)*], *cimEx6*[*P_myo-3_ctn-1*, *P_myo-3_*GFP, *ofm-1::*GFP] and *cimEx7*[*P_H20_ctn-1*, *P_H20_*GFP,*ofm-1::*GFP].

### Genetic screen for suppressor mutants of *slo-1(ky399)*

Gain-of-function *slo-1(ky399)* mutants were mutagenized by exposure to 50 mM EMS (ethane methyl sulfonate) for 4 h [43]. Suppressors that suppress or ameliorate the sluggish locomotory phenotype *of slo-1(ky399gf)* mutants were selected from F2 progeny of the mutagenized animals. We screened approximately 5,000 haploid genome size for suppressor mutants and identified a total of 17 suppressor mutants. Genetic analysis of these suppressor mutants indicates that three of these have a second mutation in the *slo-1* gene. In addition, we found that eight have mutations in genes causing head-bending phenotype (2 alleles of *dyb-1*, 3 alleles of *stn-1* and 2 alleles of *ctn-1*). The remaining six mutants do not exhibit distinct locomotory phenotypes when segregated from *slo-1(gf)*.

### Genetic mapping and cloning

For genetic mapping, *slo-1(ky399)* mutants were outcrossed 12 times to the CB4856 strain. The resulting strain was used for SNP (single nucleotide polymorphism) mapping [44]. Alternatively, we used CB4856 as a mapping strain when mapping is based on the head-bending phenotype. For transgenic rescue, fosmid clones purchased from Gene services Inc. (Cambridge, UK) were injected into the gonad of *ctn-1* mutant at 2 ng/µl along with *ofm-1*::GFP marker (30 ng/µl). Once we rescued the head bending phenotype of *ctn-1* with a single fosmid, we rescued *ctn-1* mutant with a genomic DNA fragment encompassing the entire coding sequence of *ctn-1* and approximately 4 kb upstream of the putative translation site.

To verify the predicted coding sequence of *ctn-1*, we first performed BLAST search analysis using the genomic sequences of *C. briggsae* and *C. remanei*. This analysis suggested that the first and 12th exons are longer than predicted in WormBase (WS208), and that an additional exon (10^th^ exon) is present. Second, we sequenced *C. elegans* ORF *ctn-1* clone (9349620) and confirmed the 10th and the 12th exon sequences. Third, we performed sequence analysis of the DNA fragment obtained from RT-PCR with a primer set (SL1 and an internal primer) and identified the trans-splicing site which is 29 bp upstream of the newly-defined translation initiation site. Our analysis indicates that *ctn-1* encodes a predicted protein with 784 amino acids (Figure S1B).

### Constructs and transformation

The *ctn-1* genomic DNA (approximately 4 kb upstream of the promoter and the entire coding sequence) was amplified by the expand long template PCR system (Roche Applied Science) and used directly for rescue. For P*_H20_ctn-1* and P*_myo-3_ctn-1* constructs, the neuron-specific *H20* [45] or muscle-specific *myo-3* promoter sequences were fused to the translation initiation site of the *ctn-1* genomic DNA in frame by the overlapping extension PCR (Roche). For localization of CTN-1, we inserted the GFP sequence to the translation initiation site of *ctn-1* cDNA, and then the *ctn-1* promoter sequence was inserted before the GFP sequence. The resulting construct rescued the head-bending phenotype of *ctn-1* mutants and was used for generating integrated transgenic animals. For GFP::*sgca-1* construct, the GFP sequence was inserted in-frame right after the signal sequence of *sgca-1* open-reading frame as described previously [21]. Transgenic strains were made as described [46] by injecting DNA constructs (2–10 ng/µl) along with a co-injection marker DNA (pRF4(*rol-6*(*d*)) or *ofm-1*::GFP) into the gonad of hermaphrodite animals at 100 ng/µl. We obtained at least 3 independent transgenic lines for rescue, and found that all lines show similar results.

### Measurement of locomotory speed

To remove bacteria attached to animals, approximately fifteen age-matched (30 hr after L4 stage) hermaphrodite animals for each genotype were placed on a NGM (nematode growth medium) agar plate without bacteria for 15 min. The animals were then placed inside one of two copper rings embedded in a NGM plate. We found that age of agar plate influences the speed of animals, probably because the surface tension resulting from the liquid surrounding animals slows down movement. We used approximately one week-old plates for our assay, and compared with the speed of wild-type control animals. Video frames from two different genotypes were simultaneously acquired with a dissecting microscope equipped with Go-3 digital camera (QImaging) for 2 min with a 500 ms interval and 20 ms exposure. We measured the average speed of animals by using Track Objects from ImagePro Plus (Media Cybernetics).

### Measurement of the number of eggs

The activity of egg laying muscle was measured indirectly by counting eggs retained in uteri. Single age-matched (30 hrs post-L4) animals (total 15 for each genotype) were placed in each well of a 96 well plate that contains 1% alkaline hypochlorite solution. The eggshells protect embryos from dissolution by alkaline hypochlorite. After 15 min incubation, the remaining eggs were counted in each well.

### Body curvature analysis

Body curvature analysis was previously described [47]. A single animal was transferred to an agar plate and its movement was recorded at 20 frames per second. We limited image acquisition within 15 to 60 seconds after transfer, because the head bending phenotype is prominent when animals are stimulated to move forward rapidly. A custom-written software automatically recognizes the animal and assigns thirteen points spaced equally from the tip of nose to the tail along the midline of the body, and produces the pixel coordinates of thirteen points. First supplementary angles were calculated from the coordinates of the first three points with MATLAB software. First angle data were obtained when the head swing of an animal reached the maximal extension to the dorsoventral side.

### Western blot analysis

Mixed stage worms were washed and collected in M9 buffer. Equal volume of 2× Laemmli sample buffer was added to the worm pellets. The resulting worm suspension was heated at 90 °C for 10 min, centrifuged at 20,000 g for 10 min, and then immediately loaded on 7.5% SDS-PAGE gel. The Western blot analysis was performed using anti-GFP antibody (Clontech, JL-8) and anti-α-tubulin antibody (Developmental hybridoma bank, AA4.3).

### Microscopy imaging

Fixation and immunostaining procedures are previously described [21]. Fluorescence images were observed under a Zeiss Axio Observer microscope with 40× objective (water-immersion, NA: 1.2) or an Olympus Fluoview 300 confocal microscope with a 60× objective (oil-immersion, NA: 1.4) or 100× objective (oil-immersion, NA: 1.4). We typically observed more than 50 animals for each genotype. Images for quantification were acquired under an identical exposure time, gains and pinhole diameter. The intensity of puncta from acquired images was analyzed using linescan (Metamorph, Molecular Devices) and presented as values obtained by subtracting background levels from the peak grey levels of puncta.

### Electrophysiological recordings

Electrophysiological methods were as previously described [48]. Briefly, animals raised on 80 µM retinal plates, were immobilized with cyanoacrylic glue and a lateral cuticle incision was made to expose the ventral medial body wall muscles. Muscle recordings were made in the whole-cell voltage-clamp configuration (holding potential −60 mV) using an EPC-10 patch-clamp amplifier and digitized at 2.9 kHz. The extracellular solution consisted of (in mM): NaCl 150; KCl 5; CaCl_2_ 5; MgCl_2_ 4, glucose 10; sucrose 5; HEPES 15 (pH 7.3, ∼340mOsm). The patch pipette was filled with (in mM): KCl 120; KOH 20; MgCl_2_ 4; (*N*-tris[Hydroxymethyl] methyl-2-aminoethane-sulfonic acid) 5; CaCl_2_ 0.25; Na_2_ATP 4; sucrose 36; EGTA 5 (pH 7.2, ∼315mOsm). All of the animals carry a transgene (*zxIs6*) that expresses channelrhodopsin-2 under the control of the cholinergic motor neuron (*unc-17*)-specific promoter. Evoked currents were recorded in a body-wall muscle after eliciting neurotransmitter release by a 10 ms illumination using a 470 nm LED (Thor labs) triggered with a TTL pulse from the EPC10 pulse generator [31]. Evoked post-synaptic responses were acquired using Pulse software (HEKA) run on a Dell computer. Subsequent analysis and graphing was performed using Pulsefit (HEKA), Mini analysis (Synaptosoft Inc) and Igor Pro (Wavemetrics). The data were analyzed with one-way ANOVA followed by Dunnett's multiple comparison.

## Supporting Information

Figure S1 Genetic mapping and cloning of *ctn-1*. (A) SNP used for mapping is indicated on top. The fosmid clones used for rescue experiments are listed. (B) The predicted amino acid sequence of *ctn-1*. The mutation sites within predicted amino acid sequence of *ctn-1* are indicated as bold. Overall identity of CTN-1 to human α-catulin is 39.4%. The parts of CTN-1 amino acid sequence exhibiting identity to human vinculin are underlined. (C) Muscle specific expression of *ctn-1* rescues the head bending phenotype of *cim6* mutants. First angles of body curvature are shown from different genotypes of animals. ns represents no significant difference (*P*>0.05).
(0.78 MB TIF)

Figure S2 GFP::SLO-1 expression is detected in neuronal cell bodies, and its expression levels are not altered in wild-type and *ctn-1* mutant animals. (A) GFP::SLO-1 expression near cell body of neurons. Arrows indicate right and left side of the ventral nerve cord. Arrowheads indicate patched expressions of GFP::SLO-1 near cell body. Scale bar, 10 µm. (B) The expression of SLO-1::GFP was not altered in wild-type and mutant animals. Wild-type animals without the SLO-1::GFP transgene (*control*), and wild-type (*wild-type*), *dys-1* (*dys-1*) or *ctn-1* (*ctn-1*) animals with the *slo-1*::GFP transgene were used for Western blot analysis (*WB*).
(1.44 MB TIF)

Figure S3 *ctn-1* is aldicarb sensitive, and has a neuronal function. (A) The aldicarb sensitivity of *slo-1(if)* and *ctn-1*. Twenty age-matched animals in triplicate were placed on a plate containing 0.5 mM aldicarb, and their paralysis was scored over a three-hour period. Error bars represent s. e. m. Asterisk indicates significant difference between two groups (*P*<0.05). (B) Muscle expression of *ctn-1* does not rescue prolonged synaptic responses of the *ctn-1* mutant. Wild-type (n = 31), *ctn-1* (n = 16), *ctn-1;zxIs6*;Ex[P*_myo_*_-*3*_ *ctn-1*, P*_myo_*_-*3*_GFP, *ofm-1*::GFP] (n = 7). Asterisks indicate significant difference whereas ns represents no significant difference (*P*<0.05).
(0.72 MB TIF)

Video S1 Locomotory behavior of *slo-1(ky399gf)* and *slo-1(ky399);eg1167*. A movie from *slo-1(ky399gf)* (Left side) and *slo-1(ky399);eg1167* (Right side). Mutation in *ctn-1* suppresses the sluggish movement of *slo-1(ky399gf)* animals.
(1.75 MB MOV)

## References

[1] Lai HC, Jan LY (2006). The distribution and targeting of neuronal voltage-gated ion channels.. Nat Rev Neurosci.

[2] Levitan IB (2006). Signaling protein complexes associated with neuronal ion channels.. Nat Neurosci.

[3] Salkoff L, Butler A, Ferreira G, Santi C, Wei A (2006). High-conductance potassium channels of the SLO family.. Nat Rev Neurosci.

[4] Fakler B, Adelman JP (2008). Control of K(Ca) channels by calcium nano/microdomains.. Neuron.

[5] Brenner R, Perez GJ, Bonev AD, Eckman DM, Kosek JC (2000). Vasoregulation by the beta1 subunit of the calcium-activated potassium channel.. Nature.

[6] Lovell PV, McCobb DP (2001). Pituitary control of BK potassium channel function and intrinsic firing properties of adrenal chromaffin cells.. J Neurosci.

[7] Werner ME, Zvara P, Meredith AL, Aldrich RW, Nelson MT (2005). Erectile dysfunction in mice lacking the large-conductance calcium-activated potassium (BK) channel.. J Physiol.

[8] Du W, Bautista JF, Yang H, Diez-Sampedro A, You SA (2005). Calcium-sensitive potassium channelopathy in human epilepsy and paroxysmal movement disorder.. Nat Genet.

[9] Shruti S, Clem RL, Barth AL (2008). A seizure-induced gain-of-function in BK channels is associated with elevated firing activity in neocortical pyramidal neurons.. Neurobiol Dis.

[10] Schopperle WM, Holmqvist MH, Zhou Y, Wang J, Wang Z (1998). Slob, a novel protein that interacts with the Slowpoke calcium-dependent potassium channel.. Neuron.

[11] Lu R, Alioua A, Kumar Y, Eghbali M, Stefani E (2006). MaxiK channel partners: physiological impact.. J Physiol.

[12] Tian L, Chen L, McClafferty H, Sailer CA, Ruth P (2006). A noncanonical SH3 domain binding motif links BK channels to the actin cytoskeleton via the SH3 adapter cortactin.. FASEB J.

[13] Kim EY, Ridgway LD, Dryer SE (2007). Interactions with filamin A stimulate surface expression of large-conductance Ca^2+^-activated K^+^ channels in the absence of direct actin binding.. Mol Pharmacol.

[14] Park SM, Liu G, Kubal A, Fury M, Cao L (2004). Direct interaction between BKCa potassium channel and microtubule-associated protein 1A.. FEBS Lett.

[15] Wang ZW, Saifee O, Nonet ML, Salkoff L (2001). SLO-1 potassium channels control quantal content of neurotransmitter release at the *C. elegans* neuromuscular junction.. Neuron.

[16] Davies AG, Pierce-Shimomura JT, Kim H, VanHoven MK, Thiele TR (2003). A central role of the BK potassium channel in behavioral responses to ethanol in *C. elegans*.. Cell.

[17] Carre-Pierrat M, Grisoni K, Gieseler K, Mariol MC, Martin E (2006). The SLO-1 BK channel of *Caenorhabditis elegans* is critical for muscle function and is involved in dystrophin-dependent muscle dystrophy.. J Mol Biol.

[18] Kim H, Rogers MJ, Richmond JE, McIntire SL (2004). SNF-6 is an acetylcholine transporter interacting with the dystrophin complex in *Caenorhabditis elegans*.. Nature.

[19] Bessou C, Giugia JB, Franks CJ, Holden-Dye L, Segalat L (1998). Mutations in the *Caenorhabditis elegans* dystrophin-like gene *dys-1* lead to hyperactivity and suggest a link with cholinergic transmission.. Neurogenetics.

[20] Gieseler K, Bessou C, Segalat L (1999). Dystrobrevin- and dystrophin-like mutants display similar phenotypes in the nematode *Caenorhabditis elegans*.. Neurogenetics.

[21] Kim H, Pierce-Shimomura JT, Oh HJ, Johnson BE, Goodman MB (2009). The dystrophin complex controls bk channel localization and muscle activity in *Caenorhabditis elegans*.. PLoS Genet.

[22] Alderton JM, Steinhardt RA (2000). Calcium influx through calcium leak channels is responsible for the elevated levels of calcium-dependent proteolysis in dystrophic myotubes.. J Biol Chem.

[23] Janssens B, Staes K, van Roy F (1999). Human alpha-catulin, a novel alpha-catenin-like molecule with conserved genomic structure, but deviating alternative splicing.. Biochim Biophys Acta.

[24] Barstead RJ, Waterston RH (1989). The basal component of the nematode dense-body is vinculin.. J Biol Chem.

[25] Costa M, Raich W, Agbunag C, Leung B, Hardin J (1998). A putative catenin-cadherin system mediates morphogenesis of the *Caenorhabditis elegans* embryo.. J Cell Biol.

[26] Wiesner C, Winsauer G, Resch U, Hoeth M, Schmid JA (2008). Alpha-catulin, a Rho signalling component, can regulate NF-kappaB through binding to IKK-beta, and confers resistance to apoptosis.. Oncogene.

[27] Sadoulet-Puccio HM, Rajala M, Kunkel LM (1997). Dystrobrevin and dystrophin: an interaction through coiled-coil motifs.. Proc Natl Acad Sci U S A.

[28] Hu H, Shao LR, Chavoshy S, Gu N, Trieb M (2001). Presynaptic Ca^2+^-activated K^+^ channels in glutamatergic hippocampal terminals and their role in spike repolarization and regulation of transmitter release.. J Neurosci.

[29] Sailer CA, Kaufmann WA, Kogler M, Chen L, Sausbier U (2006). Immunolocalization of BK channels in hippocampal pyramidal neurons.. Eur J Neurosci.

[30] Kaufmann WA, Ferraguti F, Fukazawa Y, Kasugai Y, Shigemoto R (2009). Large-conductance calcium-activated potassium channels in purkinje cell plasma membranes are clustered at sites of hypolemmal microdomains.. J Comp Neurol.

[31] Liewald JF, Brauner M, Stephens GJ, Bouhours M, Schultheis C (2008). Optogenetic analysis of synaptic function.. Nat Methods.

[32] Gieseler K, Mariol MC, Bessou C, Migaud M, Franks CJ (2001). Molecular, genetic and physiological characterisation of dystrobrevin-like (*dyb-1*) mutants of *Caenorhabditis elegans*.. J Mol Biol.

[33] Edgerton JR, Reinhart PH (2003). Distinct contributions of small and large conductance Ca^2+^-activated K^+^ channels to rat Purkinje neuron function.. J Physiol.

[34] Prakriya M, Lingle CJ (1999). BK channel activation by brief depolarizations requires Ca^2+^ influx through L- and Q-type Ca^2+^ channels in rat chromaffin cells.. J Neurophysiol.

[35] Gracheva EO, Hadwiger G, Nonet ML, Richmond JE (2008). Direct interactions between *C. elegans* RAB-3 and Rim provide a mechanism to target vesicles to the presynaptic density.. Neurosci Lett.

[36] Saheki Y, Bargmann CI (2009). Presynaptic CaV2 calcium channel traffic requires CALF-1 and the alpha(2)delta subunit UNC-36.. Nat Neurosci.

[37] Biggar WD, Klamut HJ, Demacio PC, Stevens DJ, Ray PN (2002). Duchenne muscular dystrophy: current knowledge, treatment, and future prospects.. Clin Orthop Relat Res.

[38] Bois PR, Borgon RA, Vonrhein C, Izard T (2005). Structural dynamics of alpha-actinin-vinculin interactions.. Mol Cell Biol.

[39] Ziegler WH, Liddington RC, Critchley DR (2006). The structure and regulation of vinculin.. Trends Cell Biol.

[40] Ervasti JM (2003). Costameres: the Achilles' heel of Herculean muscle.. J Biol Chem.

[41] Ayalon G, Davis JQ, Scotland PB, Bennett V (2008). An ankyrin-based mechanism for functional organization of dystrophin and dystroglycan.. Cell.

[42] Troemel ER, Sagasti A, Bargmann CI (1999). Lateral signaling mediated by axon contact and calcium entry regulates asymmetric odorant receptor expression in *C. elegans*.. Cell.

[43] Brenner S (1974). The genetics of *Caenorhabditis elegans*.. Genetics.

[44] Wicks SR, Yeh RT, Gish WR, Waterston RH, Plasterk RH (2001). Rapid gene mapping in *Caenorhabditis elegans* using a high density polymorphism map.. Nat Genet.

[45] Shioi G, Shoji M, Nakamura M, Ishihara T, Katsura I (2001). Mutations affecting nerve attachment of *Caenorhabditis elegans*.. Genetics.

[46] Mello CC, Kramer JM, Stinchcomb D, Ambros V (1991). Efficient gene transfer in *C. elegans*: extrachromosomal maintenance and integration of transforming sequences.. Embo J.

[47] Pierce-Shimomura JT, Chen BL, Mun JJ, Ho R, Sarkis R (2008). Genetic analysis of crawling and swimming locomotory patterns in *C. elegans*.. Proc Natl Acad Sci U S A.

[48] Richmond JE (2006). Electrophysiological recordings from the neuromuscular junction of *C. elegans*.. WormBook.

