# Supplementary figures and images for: An Alpha-Catulin Homologue Controls Neuromuscular Function through Localization of the Dystrophin Complex and BK Channels in Caenorhabditis elegans
Source: PLoS Genet. 2010 Aug 26;6(8):e1001077. doi: 10.1371/journal.pgen.1001077 (PMC2928805; doi:10.1371/journal.pgen.1001077)

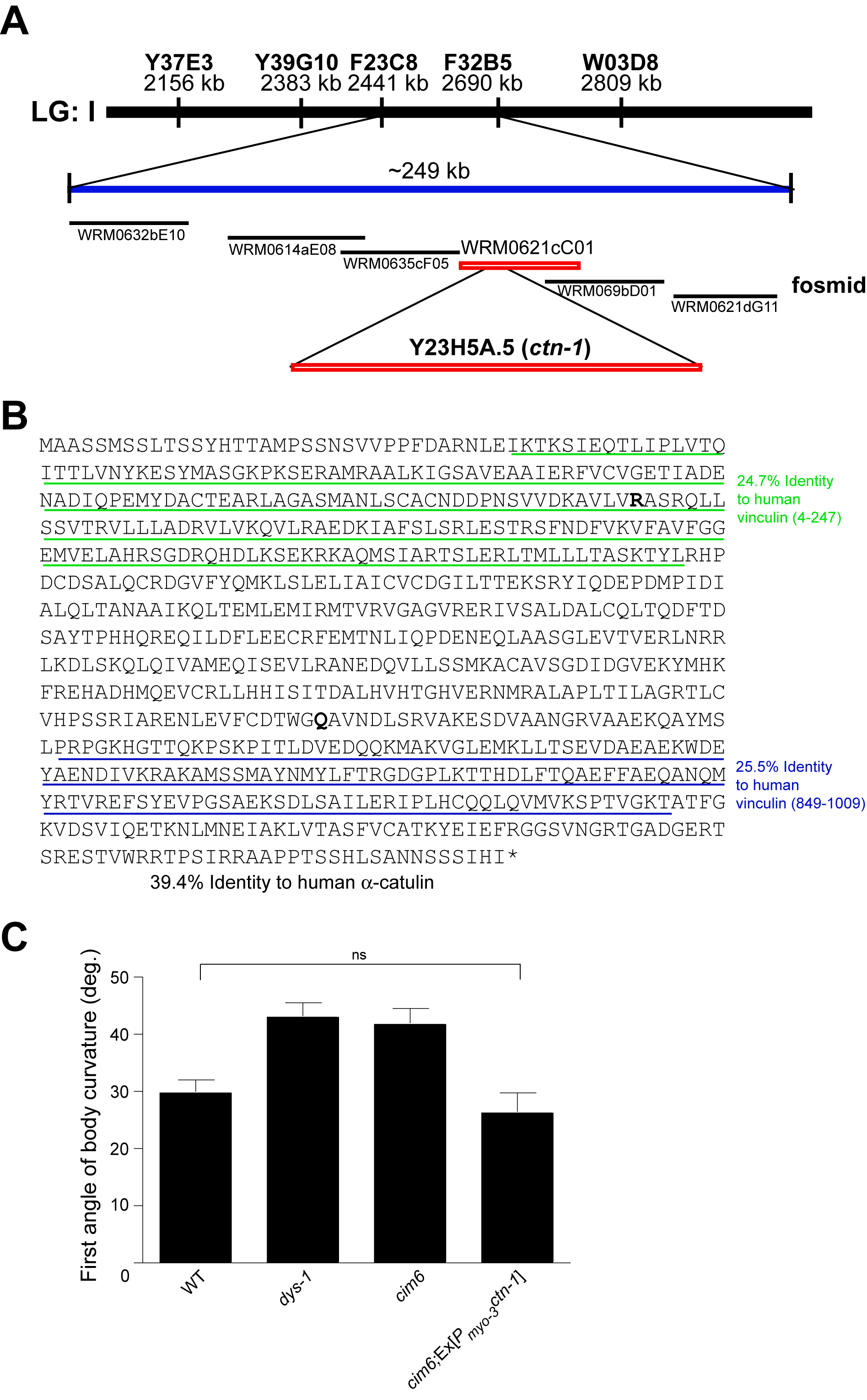

Supplement: Figure S1 — Genetic mapping and cloning of ctn-1. (A) SNP used for mapping is indicated on top. The fosmid clones used for rescue experiments are listed. (B) The predicted amino acid sequence of ctn-1. The mutation sites within predicted amino acid sequence of ctn-1 are indicated as bold. Overall identity of CTN-1 to human α-catulin is 39.4%. The parts of CTN-1 amino acid sequence exhibiting identity to human vinculin are underlined. (C) Muscle specific expression of ctn-1 rescues the head bending phenotype of cim6 mutants. First angles of body curvature are shown from different genotypes of animals. ns represents no significant difference (P>0.05). (0.78 MB TIF) [file pgen.1001077.s001.tif]

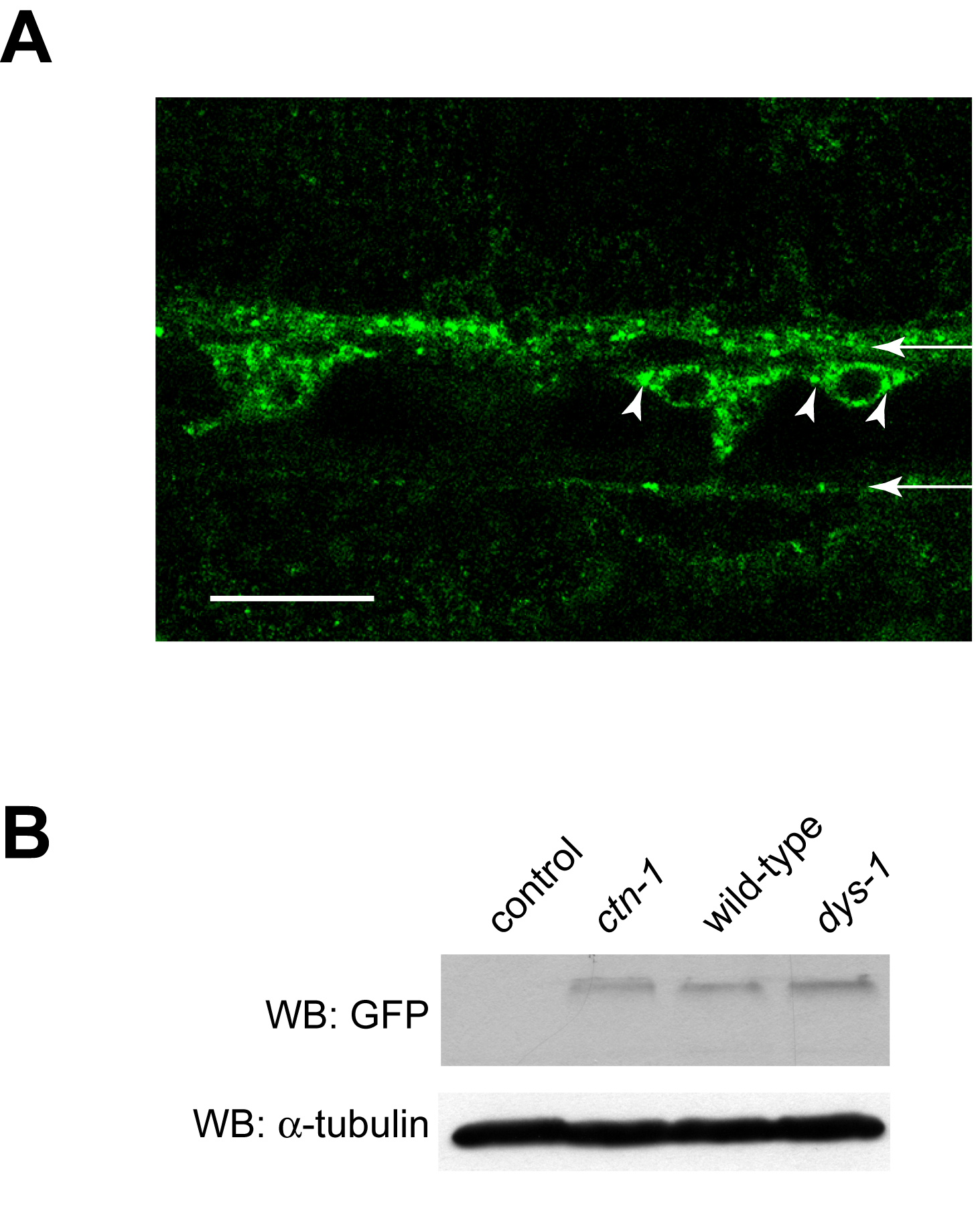

Supplement: Figure S2 — GFP::SLO-1 expression is detected in neuronal cell bodies, and its expression levels are not altered in wild-type and ctn-1 mutant animals. (A) GFP::SLO-1 expression near cell body of neurons. Arrows indicate right and left side of the ventral nerve cord. Arrowheads indicate patched expressions of GFP::SLO-1 near cell body. Scale bar, 10 µm. (B) The expression of SLO-1::GFP was not altered in wild-type and mutant animals. Wild-type animals without the SLO-1::GFP transgene (control), and wild-type (wild-type), dys-1 (dys-1) or ctn-1 (ctn-1) animals with the slo-1::GFP transgene were used for Western blot analysis (WB). (1.44 MB TIF) [file pgen.1001077.s002.tif]

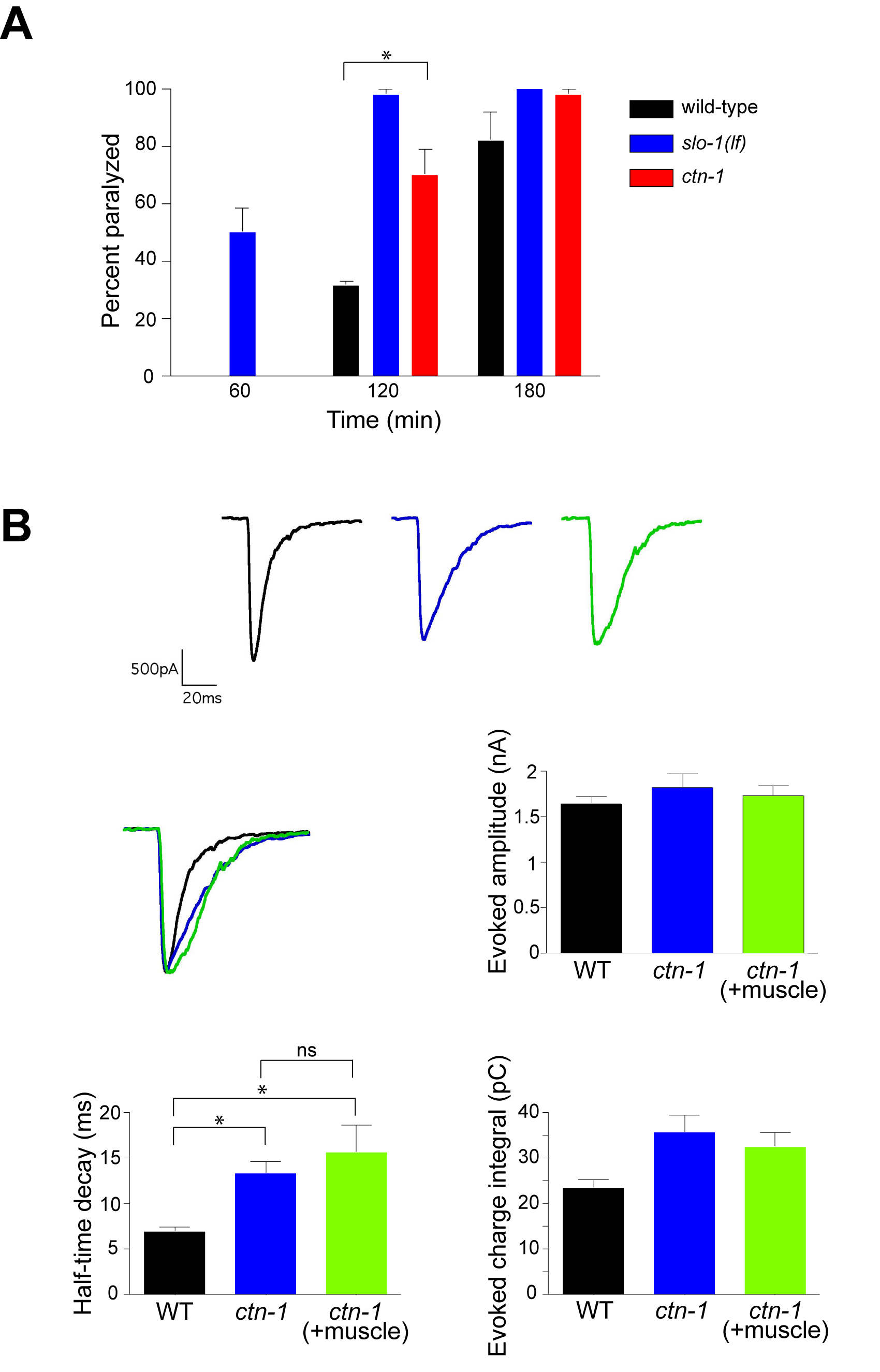

Supplement: Figure S3 — ctn-1 is aldicarb sensitive, and has a neuronal function. (A) The aldicarb sensitivity of slo-1(if) and ctn-1. Twenty age-matched animals in triplicate were placed on a plate containing 0.5 mM aldicarb, and their paralysis was scored over a three-hour period. Error bars represent s. e. m. Asterisk indicates significant difference between two groups (P<0.05). (B) Muscle expression of ctn-1 does not rescue prolonged synaptic responses of the ctn-1 mutant. Wild-type (n = 31), ctn-1 (n = 16), ctn-1;zxIs6;Ex[Pmyo -3 ctn-1, Pmyo -3GFP, ofm-1::GFP] (n = 7). Asterisks indicate significant difference whereas ns represents no significant difference (P<0.05). (0.72 MB TIF) [file pgen.1001077.s003.tif]
